# CDK7 inhibitors as anticancer drugs

**DOI:** 10.1007/s10555-020-09885-8

**Published:** 2020-05-08

**Authors:** Georgina P. Sava, Hailing Fan, R. Charles Coombes, Lakjaya Buluwela, Simak Ali

**Affiliations:** Division of Cancer, Department of Surgery & Cancer, Imperial College London, Hammersmith Hospital Campus, London, UK

**Keywords:** CDK7, CDK inhibitors, Cell cycle, Transcription, Cancer therapy, Combination therapy

## Abstract

Cyclin-dependent kinase 7 (CDK7), along with cyclin H and MAT1, forms the CDK-activating complex (CAK), which directs progression through the cell cycle *via* T-loop phosphorylation of cell cycle CDKs. CAK is also a component of the general transcription factor, TFIIH. CDK7-mediated phosphorylation of RNA polymerase II (Pol II) at active gene promoters permits transcription. Cell cycle dysregulation is an established hallmark of cancer, and aberrant control of transcriptional processes, through diverse mechanisms, is also common in many cancers. Furthermore, CDK7 levels are elevated in a number of cancer types and are associated with clinical outcomes, suggestive of greater dependence on CDK7 activity, compared with normal tissues. These findings identify CDK7 as a cancer therapeutic target, and several recent publications report selective CDK7 inhibitors (CDK7i) with activity against diverse cancer types. Preclinical studies have shown that CDK7i cause cell cycle arrest, apoptosis and repression of transcription, particularly of super-enhancer-associated genes in cancer, and have demonstrated their potential for overcoming resistance to cancer treatments. Moreover, combinations of CDK7i with other targeted cancer therapies, including BET inhibitors, BCL2 inhibitors and hormone therapies, have shown efficacy in model systems. Four CDK7i, ICEC0942 (CT7001), SY-1365, SY-5609 and LY3405105, have now progressed to Phase I/II clinical trials. Here we describe the work that has led to the development of selective CDK7i, the current status of the most advanced clinical candidates, and discuss their potential importance as cancer therapeutics, both as monotherapies and in combination settings. ClinicalTrials.gov Identifiers: NCT03363893; NCT03134638; NCT04247126; NCT03770494.

## Introduction

**Fig. 1:**
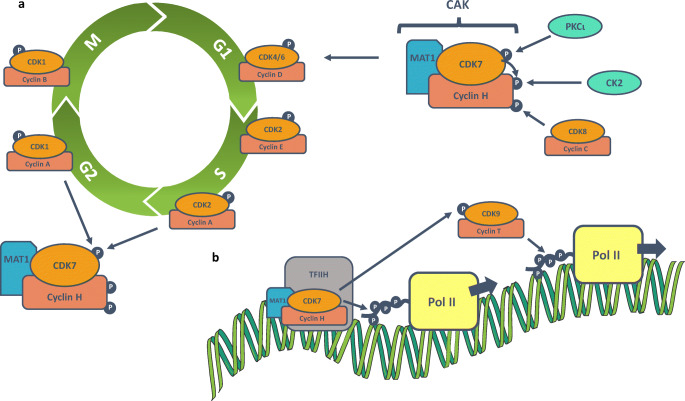
Overview of the regulation of CAK and the role of CDK7 in regulating the cell cycle (**a**) and transcription (**b**). CAK = CDK activating kinase, CDK = cyclin-dependent kinase, CK2 = protein kinase CK2, G1 = gap phase 1, G2 = gap phase 2, M = mitosis, P = phosphate, PKCι = protein kinase C iota, Pol II = RNA polymerase II, S = synthesis, TFIIH = transcription factor II H

Cyclin-dependent kinase 7 (CDK7), along with cyclin H and MAT1, comprises the CDK-activating kinase (CAK), which provides the T-loop phosphorylation required for activation of CDKs 1,2, 4 and 6, which drive cell cycle progression (Table 1, Fig. 1a) [1–4]. CAK also has a role in the regulation of transcription, as a component of the general transcription factor TFIIH. At active gene promoters, CDK7 phosphorylates the C-terminal domain (CTD) of RNA polymerase II (Pol II), at serine 5 (Ser5), to facilitate transcription initiation (Table 1, Fig. 1b) [5–7]. CDK7 also phosphorylates CDK9, which in turn phosphorylates the Pol II CTD at Ser2, to drive transcription elongation [8]. The activities of a variety of transcription factors, including p53 [9, 10], retinoic acid receptor [11–13], oestrogen receptor [14, 15] and androgen receptor [16, 17], are also regulated by CDK7-mediated phosphorylation (Table 1).

**Table 1:** CDK7 substrates

	Substrate	Residue(s)	Possible role(s)	Refs
Cell cycle	CDK1	Threonine 161	T-loop activation and cyclin binding	[1, 2]
	CDK2	Threonine 160	T-loop activation	[1]
	CDK4	Threonine 172	T-loop activation	[3]
	CDK6	Threonine 177	T-loop activation	[3]
	CDK9	Threonine 186	T-loop activation	[4]
Basal transcription	RNA Pol II	Serine 5 and Serine 7	Transcription initiation (Ser5); Unknown (Ser7)	[5–7]
	TFIIB	Serine 65	Promotion of transcription	[8]
	MED1	Threonine 1457	Recruitment to chromatin	[9]
Transcription factors	AR	Serine 515	Activation and turnover	[10, 11]
	E2F1	Serine 403 and Threonine 433	Degradation	[12]
	ER⍺	Serine 118	Activation and turnover	[13, 14]
	Ets1	Threonine 38	Recruitment of coactivators	[15]
	p53	Serine 33 and a residue between 311 and 393	Enhanced DNA binding (Ser33)	[16, 17]
	PPAR⍺	Serine 112	Activation	[18]
	PPARγ2	Serine 12/21	Activation	[18]
	RAR⍺	Serine 77	Activation	[19, 20]
	RARγ	Serine 77/79	Activation	[21]
	YAP/TAZ	Serine 128/90	Prevention of degradation	[22]

Because of its dual role in regulating the cell cycle and transcription, CDK7 has been studied as an anticancer drug target, and a number of selective inhibitors of CDK7 have been developed and investigated as cancer therapies. Preclinical studies have revealed that cancer cells can be preferentially targeted by transcriptional inhibition, at least in part because they are more reliant than normal cells on high levels of super-enhancer (SE)-driven transcription [18, 19] mediated by specific oncogenic drivers, such as RUNX1 in acute lymphoblastic lymphoma (ALL) [20] and N-MYC in neuroblastoma [21]. To date, four selective CDK7 inhibitors, ICEC0942 [22], SY-1365 [23], SY-5609 [24, 25] and LY340515 [26], have progressed to Phase I/II clinical trial for the treatment of advanced solid malignancies.

In this review we outline the role of CDK7 in both normal and tumour cells and the rationale for inhibiting CDK7 in cancer. We also discuss the development of selective CDK7 inhibitors, their mechanism of action in cancer and their potential for use in combination therapies.

## CDK7 function

### CAK structure and regulation

CDK7 is a 346 amino acid kinase, having a predicted molecular mass of 39 kDa, with an N-terminal cyclin H-binding region and a C-terminal MAT1 binding region [27]. A single crystal structure has been reported for CDK7 bound to ATP, in the inactive conformation, the structure being similar to that of the inactive conformation of ATP-bound CDK2 [28]. Cyclin H binding is obligatory for CDK7 kinase activity, whilst the addition of MAT1 stabilises the trimeric CAK complex and anchors it to TFIIH [27]. In addition, cyclin H and MAT1 binding have been shown to regulate CDK7 substrate specificity, with the trimeric CDK7-cyclin H-MAT1 complex having greater kinase activity for Pol II, in comparison to CDK7-cyclin H, which preferentially phosphorylates CDK2 [27–29].

The T-loop of CDK7 can be phosphorylated at two positions, threonine 170 (Thr170) and Ser164, enhancing both its kinase activity and ability to bind cyclin H [6]. Furthermore, T-loop phosphorylation of CDK7 seems to direct substrate specificity, with Thr170 phosphorylation stimulating activity towards Pol II over CDK2 [29]. *In vitro,* CDK1 and CDK2 can phosphorylate CDK7 and as substrates of CDK7 themselves; this hints at the possibility of a reinforcement activation loop between these CDKs [30]. In addition, protein kinase C iota (PKCι), acting downstream of PI3K signalling, can phosphorylate CDK7 at Thr170 (Fig. 1a) [31–35].

Regulation of CAK activity may also be mediated through phosphorylation of cyclin H. CK2 can activate CAK *in vitro*, *via* phosphorylation of cyclin H at Thr315 (Fig. 1a) [36], whereas CDK8 has been shown to negatively regulate transcription initiation, *via* phosphorylation of cyclin H at Ser5 and Ser304 (Fig. 1a) [37]. Furthermore, CDK7 complexed with cyclin H and/or the trimeric CAK can phosphorylate cyclin H *in vitro*. This autophosphorylation reduces activity of CDK7-cyclin H but has no apparent effect on CDK7-cyclin H-MAT1 activity. This suggests that MAT1 binding aids maintenance of the transcriptional activity of CAK by preventing regulation by cyclin H phosphorylation [28].

An additional means of CDK7 regulation has been observed in mouse neural progenitor cells, where the microRNA (miRNA) miR-210 regulates cell cycle progression by modulating expression levels of CDK7 [38]. This raises the possibility that there may be additional miRNAs that regulate CAK expression and activity in other cellular contexts. There is clearly more to be discovered with regard to the regulation of CDK7 and CAK activity and the identification of players acting upstream of CDK7 could potentially provide additional means by which to manipulate CDK7 activity.

### CDK7 in the cell cycle

CDK7 controls the cell cycle by phosphorylating the cell cycle CDKs 1, 2, 4 and 6 in their T-loops, to promote their activities (Fig. 1a) [1]. Both CDK1 and CDK2 are activated by CDK7-mediated T-loop phosphorylation, at Thr161 and Thr160, respectively (Table 1) [2, 20–22, 39]. Inhibiting CDK7 during G1 prevents CDK2 activation and delays S phase, whilst inhibition of CDK7 during S/G2 prevents CDK1 activation and mitotic entry [2, 22]. Whilst CDK7 can phosphorylate CDK2 prior to its binding to cyclin, and is not strictly required for the formation of CDK2-cyclin complexes, CDK7 phosphorylates CDK1 in concert with cyclin B binding and is required for the stabilisation of CDK1-cyclin B complexes [2, 40].

Full commitment to the cell cycle is controlled at the restriction point, through phosphorylation of retinoblastoma (RB) by CDK4/6-cyclin D, in response to mitogens (Fig. 1a). CDK7 phosphorylates both CDK4 and CDK6 in their T-loops, at Thr172 and Thr177 (Table 1), respectively, and CDK7 inhibition prevents their RB kinase activity, halting G1 progression [3, 4]. Although expression levels of the CAK components remain constant throughout the cell cycle, T-loop phosphorylation of CDK7 increases when cells are released from serum starvation [3]. Therefore, a mitogen-induced cascade of CDK T-loop phosphorylation regulates progression through G1 [3].

Unlike cyclin-bound CDK2, which remains phosphorylated for up to 12 hours after CDK7 inhibition, CDK4 and CDK6 activity is rapidly lost following CDK7 inhibition [3]. This difference is likely due to structural differences between the complexes; the T-loop of CDK2 is protected from dephosphorylation by cyclin binding, whereas the T-loops of cyclin D-bound CDK4/6 remain exposed to phosphatases [3]. As a result, CDK7 activity is required to maintain CDK4/6 activity during G1 whilst being required only for initial activation of CDK1 and CDK2 during S/G2 [3].

### CDK7 in transcription

CDK7 regulates gene expression, as a component of the general transcription factor complex, TFIIH (Fig. 1b). TFIIH is composed of two distinct sub-complexes: the core complex, which contains two DNA helicases, xeroderma pigmentosum type B (XPB) and xeroderma pigmentosum type D (XPD), along with five other structural and regulatory proteins, and the CAK complex. CAK is recruited to the core TFIIH complex *via* a reversible interaction between the ARCH domain of XPD and the latch domain of MAT1 [41, 42]. TFIIH is recruited by TFIIE to active gene promoters, where it joins the other assembled general transcription factors (TFs), and Pol II, in the preinitiation complex (PIC) [27]. The composition of TFIIH and the structure of the PIC have recently been reviewed by Rimel and Taatjes [27].

After DNA is unwound at the transcription start site (TSS) by XPB [43], Pol II must be released from the PIC to initiate transcription, in a CDK7-regulated process termed promoter escape [5]. The CTD of mammalian RPB1, the largest subunit of Pol II, contains 52 repeats of a heptad sequence, conforming to the consensus Y1-S2-P3-T4-S5-P6-S7, the residues of which can be sequentially phosphorylated to regulate Pol II activity throughout the transcription cycle [44]. Whilst unphosphorylated, Pol II remains anchored to the PIC, *via* an interaction with the mediator complex (another PIC component) [5]. CDK7 phosphorylates Ser5 and Ser7 of the Pol II CTD at gene promoters [6, 7]; Ser5 phosphorylation facilitates the release of Pol II from mediator, allowing Pol II to escape the PIC and initiate transcription (Table 1, Fig. 1b) [5, 45]. The precise function of CDK7-directed Ser7 phosphorylation is as yet unclear, but evidence suggests that Ser7 phosphorylation may promote the transcription and post-transcriptional processing of small nuclear RNA transcripts, by facilitating an interaction between the integrator complex and Pol II [46].

After promoter escape, Pol II generally generates a transcript of around 20–80 bases, before halting progress, in a process known as promoter-proximal pausing, which likely functions as a checkpoint to ensure the establishment of a range of co-transcriptional processes [6, 47]. CDK7 is required for the recruitment of two complexes, the DRB sensitivity inducing factor (DSIF) and the negative elongation factor (NELF), both of which are required to establish the promoter-proximal pause [6, 8, 48–50]. For the release of paused Pol II and commencement of the productive elongation phase of transcription, the activity of CDK9, as a component of the positive transcription elongation factor (P-TEFb), is required [8]. Like the cell cycle CDKs, for full functionality, CDK9 must undergo T-loop phosphorylation by CDK7 (Table 1, Fig. 1b) [8]. Therefore, CDK7 plays a role in both establishing the promoter-proximal pause and in release from the pause, and inhibition of CDK7 has been shown to increase the amount of Pol II paused at promoter-proximal regions [6, 51]. Active CDK9 phosphorylates the Pol II CTD, on Ser2, promoting transcriptional elongation [52]; therefore, there is an indirect requirement for CDK7 activity after Pol II pause release.

CDK7 also regulates further transcriptional processes; for example, CTD phosphorylation by CDK7 allows the co-transcriptional interaction of Pol II with enzymes that add the 5′-monomethyl-guanosine cap to nascent RNA transcripts [50]. Additionally, CDK7 is necessary for appropriate transcription termination, with read-through transcription observed upon CDK7 inhibition [6]. CDK12 and CDK13 are also involved in regulating transcription by phosphorylating the Pol II CTD during elongation [53]. *In vitro*, CDK12 can phosphorylate Ser2, Ser5 and Ser7 [54], whereas CDK13 can phosphorylate Ser2 and Ser5 [55]. Like the previously discussed CDKs, T-loop phosphorylation is necessary for CDK12/13 activation and is likely mediated by CDK7 [54, 56]; thus, it is probable that additional transcriptional substrates of CDK7, and further roles in transcriptional regulation, remain to be identified.

Genetic targeting of Mat1 or Cdk7 in mice is early embryonic lethal and cells cultured from embryos of these animals fail to enter S phase [57, 58]. The activities of Cdks 2, 4 and 6 are reduced in mouse embryonic fibroblasts (MEFs) with Cdk7 knockout, indicating that Cdk7 has an essential role in cell proliferation [58]. Cdk7 targeting in adult animals results in phenotypically normal low-proliferating tissues, such as the liver, kidney or cerebellum. However, in rapidly dividing epithelial tissues, Cdk7 expression is retained due to tissue renewal sustained by stem cells with incomplete Cdk7 knockout. This eventually leads to stem cell exhaustion and premature ageing [58]. Interestingly, MEFs lacking Cdk7 expression have unaltered Pol II CTD Ser5 phosphorylation and a largely unchanged gene expression program, indicating that Cdk7 is dispensable for *de novo* transcription [58]. This raises the possibility that another Pol II CTD kinase can compensate for a lack of Cdk7.

### CDK7 as a regulator of transcription factor activity

Alongside its critical role in directing transcription by Pol II, CDK7 phosphorylates a number of TFs, functioning to either promote their activities and/or regulate their degradation (Table 1). The activity of retinoic acid receptor ⍺ (RAR⍺) is promoted by XPD-dependent phosphorylation of Ser77 by CDK7 [11, 13]. Likewise, the activity of RARγ is also modulated by phosphorylation by TFIIH-incorporated CDK7 [12]. CDK7, as part of TFIIH, mediates ligand-dependent phosphorylation of oestrogen receptor ⍺ (ER⍺) at Ser118 [14, 15], regulating the activity and turnover of the TF [59, 60]. Phosphorylation by CDK7, at Ser515 in the transcription activation function of androgen receptor (AR), has also been reported [16, 17]. Additionally, CDK7 can phosphorylate p53 in a MAT1-dependent fashion, at both the C-terminus (between residues 311 and 393) [10] and the N-terminus, at Ser33 [9], the former of which has been shown to stimulate p53 binding to DNA. Evidence that CDK7 phosphorylates Ets1 [61], peroxisome proliferator-activated receptors (PPARs) [62] and E2F1 has also been demonstrated, the latter functioning to trigger E2F1 degradation [63] (Table 1). Recently, the stabilisation of the transcriptional regulators YAP/TAZ was shown to be mediated by CDK7, with phosphorylation of YAP at Ser128 and TAZ at Ser90, preventing their ubiquitination and degradation [64]. At present we have an incomplete understanding of the role CDK7 plays in regulating the activities of sequence-specific transcriptional regulators. Further knowledge in this area may be helpful in informing the use of CDK7 inhibitors in specific cellular contexts.

### CDK7 in DNA repair

TFIIH plays a key role in the nucleotide excision repair (NER) pathway [27], which repairs single-stranded DNA damage, particularly that caused by ultraviolet light. TFIIH is recruited to damaged DNA, where the NER protein, xeroderma pigmentosum group A (XPA), catalyses the release of CAK from the core TFIIH complex, allowing NER to proceed [65]. After DNA repair, CAK reassociates with TFIIH, and the complex resumes its role in transcription [65]. Inhibition of CDK7 kinase activity improves NER efficiency, suggesting that CDK7 negatively regulates NER, directly or indirectly, *via* phosphorylation of an as yet unidentified substrate(s) [66].

## CDK7 in cancer

### CDK7 expression in tumours

Two decades ago, immunohistochemical analyses on a range of tumour types indicated that CDK7 expression is elevated in tumour cells compared with their normal counterparts [67]. Since then, numerous studies have provided support for this finding [68–73]. In oestrogen receptor-positive (ER+) breast cancer, CDK7, cyclin H and MAT1 are overexpressed and are co-regulated at the mRNA level [68]. Expression of the CAK components positively correlates with ER expression and Ser118 phosphorylation, as well as with improved patient outcomes [68]. Conversely, in triple-negative breast cancer (TNBC), CDK7 expression is correlated with poor prognosis [74]. In addition, associations between CDK7 and reduced survival have been observed in gastric cancer [69, 70], ovarian cancer [75], oral squamous cell carcinoma (OSCC) [71], hepatocellular carcinoma [72] and glioblastoma [73]. For OSCC, animal studies have also revealed a potential role for CDK7 in disease development [71].

These findings raise the possibility that tumours with increased expression of CDK7 may be more sensitive to CDK7 inhibition, particularly in the case of ER+ breast cancer, where the CDK7-activated nuclear receptor, ER⍺, drives tumour progression.

### Transcriptional addiction in cancer

Common molecular features of cancer, such as mutation, copy number changes and genomic rearrangements, can either directly or indirectly impact gene expression profiles that drive cancer. For instance, a *BRAF* mutation in melanoma causes a cascade of signalling events that ultimately leads to an altered transcriptional profile and a distinct gene expression signature [76, 77]. Mutations in TF genes are also common in cancer [78, 79]. Across all cancer types, the most frequently mutated gene (*TP53*) encodes for the TF p53 [80], and the most frequently amplified gene, *MYC* [81], also encodes for a TF. Other TFs are critical in specific tumour types. For example, ER⍺ activity drives the majority of breast cancer, and therapies that target ER⍺, like tamoxifen [82] and fulvestrant [83] are used in the treatment of ER+ breast cancer. Mutation, rearrangements and deregulated expression of genes encoding chromatin remodelling and histone modification enzymes, such as EZH2 and ARID1A, are also frequent in cancer [78, 79]. These aberrations alter the accessibility of gene regulatory regions, ultimately leading to downstream changes in gene expression.

**Fig. 2:**
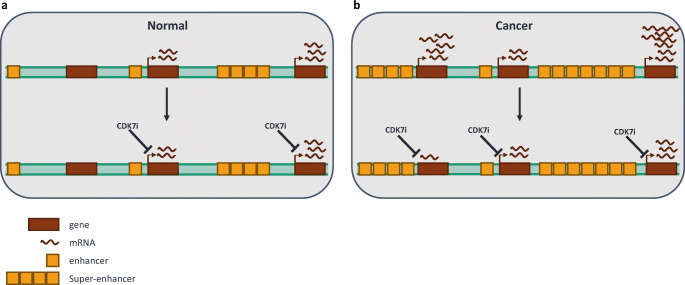
Super-enhancer-driven gene deregulation in cancer can be targeted by CDK7 inhibitors. The super-enhancer landscape in normal cells (**a**) becomes deregulated in cancer (**b**), leading to altered gene expression. CDK7 inhibitors preferentially reduce gene expression driven by super-enhancers in cancer cells compared with normal cells (A and B). CDK7i = CDK7 inhibitor

Recently, clusters of enhancers, termed super-enhancers (SE), that control the expression of genes integral for cell identity and function have been defined [84]. Deregulation of the SE landscape is common in cancer and leads to dramatic changes in gene expression and high transcriptional outputs, which maintain the oncogenic cell state (Fig. 2). As a result, cancer cells become transcriptionally addicted, requiring higher levels of transcription than normal cells to sustain growth [19]. The phenomenon of transcriptional addiction suggests that cancer cells may be more responsive than normal cells to transcriptional inhibition and provides a strong basis for targeting transcriptional kinases, including CDK7, in cancer (Fig. 2) [18]. Furthermore, oncogenic TFs, like MYC, have proven notoriously difficult to target directly with small molecules; therefore, the ability to target the general transcription machinery to reduce their transcriptional output is an attractive prospect.

## Development of CDK7 inhibitors

### Pan-CDK inhibitors

**Fig. 3:**
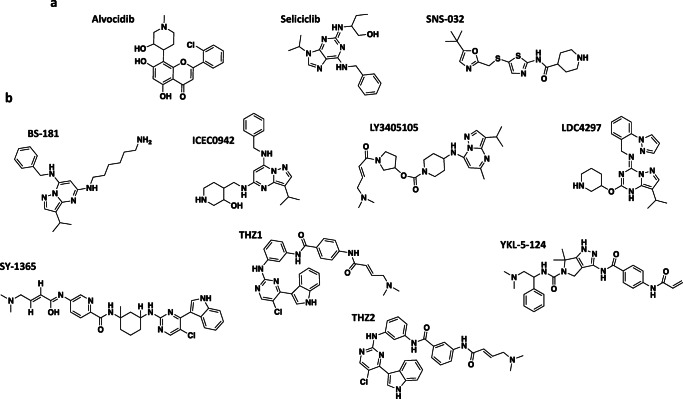
Chemical structures of selected inhibitors that target CDK7. Chemical structures of non-specific inhibitors of CDK7 (**a**) and selective inhibitors of CDK7 (**b**). (The chemical structures of QS1189 and SY-5609 have not been disclosed)

Early efforts to develop CDK inhibitors yielded relatively unselective compounds, with activities against multiple CDKs, often including CDK7 [85]. The first CDK inhibitor to enter clinical trial was the semi-synthetic flavone derivative, alvocidib (flavopiridol; Fig. 3a), which inhibits CDK1, 2, 4, 6, 7 and 9 (Table 2) [87–90]. Between 2008 and 2014, alvocidib was evaluated in more than 60 clinical trials for numerous tumour types [91]. Limited clinical activity was seen in the majority of trials, however, modest responses against chronic lymphocytic leukaemia (CLL) [92, 93] and mantle cell lymphoma [94] were shown. Currently, alvocidib, marketed as a CDK9 inhibitor, is being trialled by Tolero Pharmaceuticals for the treatment of acute myeloid leukaemia (AML) (Clinicaltrials.gov identifiers: NCT03298984; NCT03969420; NCT02520011). Another early pan-CDK inhibitor, the purine-based seliciclib (roscovitine; Fig. 3a), which inhibits CDK1, 2, 5, 7, and 9 (Table 2) [95–97], was also assessed in clinical trials for a variety of tumour types but, likewise, showed limited clinical activity [91, 98].

**Table 2:** Characteristics of selected multi-target CDK7 inhibitors (see ref [86])

Name(s)	Company^a^	IC_50_ (nM)^b^	Development phase reached
Alvocidib (flavopiridol)	Tolero Pharmaceuticals (Sanofi-Aventis)	CDK1-CycB = 41; CDK2-CycA = 100; CDK4-CycD = 65; CDK6-CycD = ~100; CDK7-CycH = ~300; CDK9-CycT = 6	Phase II
Seliciclib (roscovotine; CYC202)	Cyclacel (ManRos Therapeutics)	CDK1-CycB = 2700; CDK2-CycE = 100; CDK4-CycD1 > 10,000; CDK6-CycD1 > 100,000; CDK7-CycH = 490; CDK9-CycT = 600	Phase II
SNS-032	Sunesis (Bristol-Myers Squibb)	CDK1-CycB = 480; CDK2-CycA = 38; CDK4-CycD = 92;5 CDK6-CycD>1000; CDK7-CycH = 62; CDK9-CycT = 4	Phase I

^a^Current developer (previous developer in brackets)

^b^Data from *in vitro* kinase assays for CDKs 1, 2, 4, 6, 7, 9 and 12 have been listed, where available. Where data are available for a CDK in multiple cyclin complexes, the complex with the lowest IC_50_ is presented

Attempts to develop CDK inhibitors with improved selectivity for CDK1 and CDK2 led to a second generation of multi-target CDK inhibitors, including the aminothiazole-based compound, SNS-032 (Fig. 3a), which potently inhibits CDK2, 7 and 9 (Table 2) [85, 91, 99, 100]. Although SNS-032 has been trialled for the treatment of advanced lymphoid [101] and advanced solid malignancies [102], the drug has not progressed further than Phase I [91].

The inability of these early CDK inhibitors to selectively target individual CDK family members probably contributed to their failure in the clinic. As several CDK proteins are critical for the function of normal tissues, the promiscuity of these compounds likely limits their ability to discern cancer cells from normal cells, resulting in a narrow therapeutic window and associated toxicities, which include fatigue, diarrhoea, nausea and hyperglycaemia [91, 102–105]. In addition, their lack of specificity makes it difficult to decipher which CDKs are inhibited *in vivo*, and which are most important for their underlying mechanism of action [91]. This paucity of knowledge limits the potential to develop these pan-CDK inhibitors further as targeted therapies.

More recent efforts have focused on further improving the selectivity of CDK inhibitors, with selective CDK4/6 inhibitors proving the biggest success story to date. Three CDK4/6 inhibitors have been approved for the treatment of hormone receptor (HR)-positive metastatic breast cancer: palbociclib (PD0332991; Ibrance), ribociclib (LEE011; Kisquali) and abemaciclib (LY2835219; Verzenio), in combination with aromatase inhibitors or fulvestrant [106]. More than one hundred clinical trials for CDK4/6 inhibitors in breast, but also in other cancers, including glioma, sarcoma, lung, pancreatic, head and neck, colorectal, prostate and ovarian cancer, are actively recruiting patients or about to initiate. The success of these selective CDK4/6 inhibitors is encouraging and provides some confidence that selective inhibitors of other CDKs may prove similarly successful.

### CDK7-specific inhibitors

A number of selective small molecule inhibitors of CDK7 have been developed. These include the pyrazolopyrimidine derivatives, BS-181 [39] and ICEC0942 [22, 107], and the pyrazolotriazine derivatives, LDC4297 [108] and QS1189 [109] (Fig. 3b, Table 3). These are type I inhibitors that bind reversibly to the ATP-binding site of CDK7. ATP-competitive covalent inhibitors of CDK7 have also been developed, including the pyrimidine based THZ1 [20] and SY-1365 [23] and the pyrrolidinopyrazole based YKL-5-124 [130] (Fig. 3b, Table 3).

**Table 3:** Characteristics of key CDK7 inhibitors in development

Name(s)	Company	Type of inhibitor	IC_50_ (nM)^a^	Activity in preclinical models	Combination agents tested	Current development phase (clinical Trial ID)
BS-181 [39]	-	Non-covalent	CDK1-CycB = 8100; CDK2-CycE = 880; CDK4-CycD1 = 33,000; CDK6-CycD1 = 47,000; CDK7-CycH-Mat1 = 21; CDK9-CycT = 4200	ER+ breast cancer [39], gastric cancer [110], papillary thyroid cancer [111]	-	–
ICEC0942 [22] (CT7001)	CarrickTherapeutics	Non-covalent	CDK1-CycA1 = 1800; CDK2-CycA1 = 620; CDK4-CycD1 = 49,000; CDK6-CycD1 = 34,000; CDK7-CycH-MAT1 = 40; CDK9-CycT1 = 1200	ER+ breast cancer [22], AML [112]	Fulvestrant, tamoxifen [22]	Phase I/II (NCT03363893)
LY3405105 [26]	Eli Lilly and Company	-	CDK1-CycB1 = 20,000; CDK2-CycE1 = 20,000; CDK4-CycD1 = 2830; CDK6-CycD1 = 8079; CDK7-CycH-Mat1 = 92.8; CDK9-CycT1 = 6320; CDK12-CycK = 14,780	-	-	Phase I (NCT03770494)
LDC4297 [108]	Lead Discovery Center GmbH	Non-covalent	CDK1-CycB = 54; CDK2-CycE = 6.4; CDK4-CycD ≥ 1000; CDK6-CycD > 1000; CDK7-CycH-MAT1 < 5; CDK9-CycT = 1711	HCMV antiviral activity [113]	-	-
QS1189 [109]	Qurient Therapeutics	Non-covalent	CDK1-CycE1 = 690; CDK2-CycE1 = 270; CDK4-CycD1 = 3700; CDK6-CycD1 = 6200; CDK7-CycH-MAT1 = 15; CDK9-CycK = 710; CDK12-CycK = 570	Mantle cell lymphoma, Burkitt’s lymphoma, DLBCL [109]	-	-
SY-5609 [24]	Syros Pharmaceuticals	Non-covalent	CDK2-CycE1 = 2900^c^; CDK7-CycH-MAT1 = 0.06^b^; CDK9-CycT1 = 970^c^; CDK12-CycK = 770 nM^c^	ER+ breast cancer [25], ovarian cancer [24], TNBC [24]	Fulvestrant [25]	-
SY-1365 [23]	SyrosPharmaceuticals	Covalent	CDK2-CycE1 = 2117; CDK7-CycH-MAT1 = 84; CDK9-CycT1 = 914; CDK12-CycK = 204	AML [23]	Venetoclax [23]	Phase I (NCT03134638)
THZ1 [85] (SY-079)	Syros Pharmaceuticals	Covalent	CDK7-CycH = 3.2	T-ALL [20], neuroblastoma [21], SCLC [114], OSCC [71], PTCL [115], ovarian cancer [75], DIPG [116], HGG [117], melanoma [118], hepatocellular carcinoma [119], thyroid cancer [120], pancreatic cancer [121], cervical cancer [122], TNBC [123], multiple myeloma [124]	Fulvestrant [125], JQ1 [75, 116, 126], panobinostat [116], carfilzomib/bortezomib [124], venetoclax [124]/navitoclax [127], 5-fluorouracil, nutlin-3 [128]	-
THZ2 [123]	Syros Pharmaceuticals	Covalent	CDK1-CycB = 97; CDK2-CycA = 222; CDK7-CycH = 14; CDK9-CycT = 194	TNBC [123], gastric cancer [129]	-	-
YKL-5-124 [130]	Syros Pharmaceuticals	Covalent	CDK7-CycH-MAT1 = 9.7; CDK2-CycA = 1300; CDK9-CycT1 = 3020	Mantle cell lymphoma [130]	anti-PD-1+chemotherapy [131]	-

^a^Data from *in vitro* kinase assays for CDKs 1, 2, 4, 6, 7, 9 and 12 have been listed, where available. Where data are available for CDKs in complex with multiple cyclins, the complex with the lowest IC_50_ is presented. ^b^*K*_*d*_ determined by SPR. ^c^*K*_*i*_ determined by activity assay

The first example of a highly selective CDK7 inhibitor was BS-181, which is structurally related to the pan-CDK inhibitor roscovitine (Fig. 3b, Table 3) [39]. BS-181 reduced phosphorylation of CDK7 targets and impaired cancer cell line and xenograft tumour growth, establishing CDK7 as a putative cancer drug target [39]. Although *in vivo* activity was demonstrated, poor bioavailability and insufficient cell permeability precluded the development of BS-181 as a clinical candidate [39].

Efforts to develop BS-181 analogues which retain CDK7 selectivity, but have improved drug-like properties, led to the first orally bioavailable CDK7 inhibitor, ICEC0942 (CT7001; Fig. 3b, Table 3) [22, 107]. Although crystal structures of CDK7 bound to ICEC0942 could not be obtained, a crystal structure of CDK2 in complex with ICEC0942 was solved [107]. Using this structure as a starting point, modelling studies revealed aspartate 155 (Asp155) as a residue that is likely key in determining the selective binding of ICEC0942 to CDK7 [107]. ICEC0942 potently inhibited the growth of a panel of cancer cell lines and of ER+ breast cancer xenografts, and its favourable absorption, distribution, metabolism, and excretion (ADME) and pharmacokinetic (PK) properties made ICEC0942 a promising clinical candidate [22]. The drug was licenced to Carrick Therapeutics and is now in Phase I/II clinical trial for advanced solid malignancies, with focused cohorts of breast and prostate cancer patients (Table 4).

**Table 4:** Summary of clinical trials investigating CDK7 inhibitors in cancer

Drug name(s)	Clinical trial ID	Dates	Administration	Trial type	Trial design		No. of patients	Combination agent	Results
ICEC0942 (CT7001)	NCT03363893	Nov 2017–March 2021	Orally once daily	Modular Phase I/II	Module 1A	Dose-escalation/safety advanced solid tumours	39		MBAD = 120 mg once daily
					Module 1B	Refine dose-escalation/safety—up to 4 cohorts:			MTD = 360 mg once daily
						Locally advanced or metastatic TNBC	Up to 50		
						Castrate-resistant prostate cancer	Up to 25		
						Additional cohorts (may include ovarian and SCLC)	Up to 25		
					Module 2	Phase Ib/II safety and efficacy			
						Locally advanced or metastatic HR+HER2—breast cancer	Up to 75	Fulvestrant	
LY3405105	NCT03770494	Jan 2019–May 2022	Orally	Phase Ia/Ib		Safety advanced or metastatic solid tumours	Up to 215		
SY-1365	NCT03134638	May 2017–Nov 2019	Intravenously once/twice weekly	Phase I (2 parts)	Part 1	Dose-escalation/safety advanced solid tumours	~ 35		
					Part 2	Refine safety and test efficacy—5 cohorts:			
						Ovarian cancer treated with ≥ 3 prior lines of therapy	~ 24		
						Relapsed ovarian cancer with previous platinum therapy	~ 24	Carboplatin	
						Primary platinum refractory ovarian cancer	~ 12		
						Biopsy-accessible advanced solid tumours	20–30		
						HR+metastatic breast cancer post CDK4/6 + aromatase inhibitor treatment	~ 12	Fulvestrant	
SY-5609	NCT04247126	Jan 2020–Jun 2021	Orally	Phase I		Dose-escalation select advanced solid tumours	60		

*HER2* human epidermal growth factor receptor 2, *HR* hormone receptor, *MBAD* minimum biologically active dose, *MTD* maximum tolerated dose, *SCLC* small cell lung cancer, *TNBC* triple-negative breast cancer

A number of covalent CDK7 inhibitors have also been developed, the first being THZ1 (Fig. 3b, Table 3), which targets a cysteine residue (Cys312) on a C-terminal extension just outside the ATP-binding site of CDK7 [20, 132] and has strong activity in many cancer types (Table 3) [20, 21, 71, 75, 114–124]. However, THZ1 also covalently links to CDK12 and CDK13, at Cys1039 and Cys1017, respectively, inhibiting their activity [20, 132]. THZ1 has been widely employed as a tool to interrogate CDK7 function [50, 51]; however, it was recently shown that its anti-transcriptional and antitumour activities are reliant on inhibition of CDK12 and CDK13, in addition to CDK7 [130]. Consequently, YKL-5-124 was developed, with a strategy that combined the covalent warhead of THZ1 with the pyrrolidinopyrazole core of the PAK4 inhibitor, PF-3758309 [130]. Like THZ1, YKL-5-124 covalently links to Cys312 of CDK7 but does not affect the activities of CDK12 and 13 (Fig. 3b, Table 3) [130]. An analogue of THZ1, with altered regiochemistry of the acrylamide and increased *in vivo* stability has also been developed and was designated THZ2 (Fig. 3b, Table 3) [123].

To improve on the potency, selectivity and metabolic stability of THZ1, the THZ1-derived CDK7 inhibitor, SY-1365, was developed by Syros Pharmaceuticals as a candidate for clinical development [23]. SY-1365 entered Phase I clinical trial for the treatment of advanced solid tumours, with planned expansion cohorts focusing on ovarian cancer and breast cancer (Fig. 3b, Table 3 and 4). However, Syros Pharmaceuticals recently announced discontinuation of the clinical development of SY-1365 and the prioritisation of a new, orally available CDK7 inhibitor, SY-5609 (Table 3), with greater selectivity and potency for CDK7 [24, 25]. SY-5609 has antitumour activity in preclinical models of ovarian cancer [24, 25], TNBC [24, 25] and ER+ breast cancer, in combination with fulvestrant [25], and sustained tumour regressions were associated with alterations in the RB pathway [25]. A Phase I trial, in patients with select advanced solid tumours, began in early 2020 (Table 4).

Another CDK7 inhibitor, LY3405105, developed by Eli Lilly, is also undergoing clinical testing for advanced or metastatic solid cancers [26] (Fig. 3b, Table 3 and 4). Little information on LY3405105 has been released; however, selectivity data from the corresponding patent (WO2019099298) is listed in Table 3.

## Inhibiting CDK7 in cancer

Due to the importance of CDKs in regulating cell proliferation, and the deregulation of CDK pathways in many cancer types, CDKs have long been considered important targets for the design of cancer therapeutics [39]. The early pan-CDK inhibitors, alvocidib and seliciclib, cause cell cycle arrest and apoptosis, as well as altered expression of genes in these pathways [85]. Seliciclib was also shown to reduce Pol II CTD phosphorylation and Pol II-dependent transcription in myeloma cells [133]. Whilst it is likely that some cellular actions of these inhibitors are mediated through CDK7, their lack of selectivity made it difficult to distinguish CDK7-specific effects and ultimately led to the numerous side effects that resulted in their failure in the clinic. The recently developed, highly specific inhibitors of CDK7 have been instrumental in revealing the potential of CDK7 as a cancer drug target. Xenograft studies in mice showed that CDK7 inhibitors are well tolerated and effective at reducing tumour growth *in vivo* [21–23, 39, 114].

A number of reversible and covalent inhibitors of CDK7 have been tested on large panels of cancer cell lines [20, 22, 23, 39]. Screening of ICEC0942 against the NCI-60 cancer cell line panel demonstrated a median GI_50_ value of 250 nM [22]. Of over 1000 cancer cell lines tested with THZ1, around half had a GI_50_ value under 200 nM [20]. Whilst CDK7 inhibitors are potent at impairing the growth of many cancer cell lines, representing a variety of tumour types, it is clear that some cell lines respond more favourably than others. Responses of 386 human cell lines, encompassing 26 cancer types, to the covalent CDK7 inhibitor SY-1365, revealed varied responses ranging from cytostatic to highly cytotoxic [23]. Expression levels of the anti-apoptotic protein BCL-XL were predictive of SY-1365 response, with low BCL-XL expression associated with high SY-1365 sensitivity [133]. It is clear that additional features associated with response to CDK7 inhibition remain to be discovered and this knowledge will likely be beneficial for their future clinical success.

### Effects on cell cycle progression

As CDK7 directs cell cycle progression, *via* the activation of other CDK proteins, it is unsurprising that CDK7 inhibitors reduce phosphorylation of cell cycle CDKs and consequently cause cell cycle arrest [21, 22, 39, 108, 109, 130]. CDK7 inhibition with ICEC0942 blocks progression at all stages of the cell cycle and is associated with a reduction in phosphorylation of CDK1, CDK2 and RB [22]. YKL-5-124 primarily causes G1 arrest, with a reduction in CDK2 phosphorylation [130], whereas THZ1 and QS1189 both arrest cells at G2/M [21]. Interestingly, the extent and timing of cell cycle arrest upon treatment with an individual CDK7 inhibitor can vary among cell lines; A549 lung cancer cells, treated with LDC4297, arrested in G1, whereas HCT116 colon cancer cells exhibited a G2/M delay, only after an extended incubation period [108]. Again, it is apparent that factors influencing the effect of CDK7 inhibition on cell cycle progression across different cancers remain to be identified and these may have an important impact on the clinical use of these inhibitors. In addition to cell cycle arrest, apoptosis is observed following CDK7 inhibition, in numerous cancer types, including solid tumours [21–23, 39, 108, 114] and haematological malignancies [20, 109]. YKL-5-124 is unique among the current CDK7 inhibitors, in that it causes cell cycle arrest at both G1 and G2/M in the apparent absence of apoptosis [130].

### Effects on transcription

The majority of CDK7 inhibitors, again, with YKL-5-124 being an exception, reduce Pol II CTD phosphorylation and cause widespread alterations in Pol II-mediated transcription [20, 22, 23, 39, 108, 109, 130]. As CDK7 is dispensable for global transcription [58], it makes sense that rather than reducing gene expression globally, only subsets of genes are downregulated by CDK7 inhibitors. Pathway analyses suggest that cell cycle and DNA damage repair pathways are enriched in gene sets altered by CDK7 inhibition [23, 109, 114]. Additionally, smaller subsets of genes are actually upregulated following CDK7 inhibition [21, 108]. For example, around half of the 2% of genes whose expression was affected by short-term LDC4297 treatment were upregulated [108]. mRNAs have differing half-lives, and it has been noted that those with short half-lives are preferentially downregulated by CDK7 inhibitors [108]. One explanation for the counterintuitive observation that some genes are upregulated following CDK7 inhibition is that these represent a subset that are negatively regulated by genes whose transcripts have short half-lives. It is also possible that CDK7 plays a direct and crucial role in regulating the transcription of specific subsets of genes, possibly also repressing the expression of some genes, either in a direct or an indirect fashion. One clue that may go some way to explaining how the transcription of certain genes may escape CDK7 inhibition comes from the work of Shandilya et al. [134]. They showed that CDK7 phosphorylates the general transcription factor, TFIIB (Table 1), and this is required for the transcription of certain genes, but not for transcription of p53 target genes, which escape CDK7 inhibition.

Numerous studies have shown that SE-associated genes are preferentially downregulated in cancer cells treated with CDK7 inhibitors (Fig. 2) [20, 21, 71–73, 75, 114, 116, 118, 123, 135, 136]. In T cell acute lymphoblastic leukaemia (ALL), expression of the oncogenic TF, RUNX1, is driven by a large SE and is disproportionately repressed by THZ1 treatment [20]. Similarly, neuroblastomas driven by MYCN amplification, which promotes the formation of aberrant SEs, are selectively sensitive to CDK7 inhibition [21]. As MYC TFs are frequently upregulated in cancer but have proven difficult to target directly, blocking MYC expression and/or targeting the transcription machinery downstream of MYC, by inhibiting CDK7, is an attractive strategy. Similarly, oncogenic ETS TFs, which are also notoriously difficult to target, are reduced by THZ1 treatment in prostate cancer [137]. Other cancers in which THZ1-mediated downregulation of SE-associated genes has been demonstrated include ovarian cancer [75], melanoma [118] and small cell lung cancer [114]. These studies have been integral for uncovering the aforementioned phenomenon of transcriptional addiction in cancer (Fig. 2). Furthermore, targeting specific oncogenic transcriptional programs provides some explanation for the observation that cancer cells are more vulnerable to transcriptional inhibition than normal cells.

### CDK7 inhibitors to treat drug-resistant cancers

CDK7 inhibition represents a novel strategy to treat cancers with *de novo* or acquired resistance to other drugs, where further treatment options are limited. Mutations in the ESR1 gene are common in advanced ER+ breast cancer, causing oestrogen-independent receptor activation and hormone therapy resistance [125]. CDK7 is an essential gene in both ER-wild-type and ER-mutant breast cancer, and ER-mutant MCF7 cells, that are partially resistant to anti-oestrogens, are sensitive to CDK7 inhibition [125, 138]. Furthermore, activating Ser118 phosphorylation of mutant ER is inhibited by THZ1 [125, 138]. This suggests that CDK7 inhibitors may be effective at treating advanced, ER-mutant breast cancer. THZ1 has also been shown to overcome HER2 inhibitor resistance in breast cancer [139] and venetoclax resistance in mantle cell lymphoma [109], and to inhibit castration-resistant prostate cancer [137].

A number of CDK4/6 inhibitors are clinically approved for use in combination with endocrine therapies for the treatment of ER+ breast cancer, however, resistance to these drugs is an emerging problem [140]. Breast cancer cells with acquired resistance to palbociclib remain sensitive to THZ1 [140], suggesting that CDK7 inhibitors may be useful following the onset of resistance to drugs that target other CDKs.

Current clinical trials of CDK7 inhibitors are aimed at patients with advanced or metastatic cancer; therefore, a majority of these will have received other lines of therapy prior to their recruitment. The SY-1365 trial was designed to target specific cohorts of patients with resistance to prior treatments, including platinum resistance in ovarian cancer and CDK4/6 inhibitor plus aromatase inhibitor resistance in HR+ breast cancer (Table 4). The prospect of using CDK7 inhibitors to overcome resistance to prior treatments in cancer is exciting, and it is crucial that information garnered from preclinical studies of CDK7 inhibitors in the context of acquired drug-resistance continues to be considered during clinical trial design.

### Combination treatment strategies

Cancers are frequently treated with two or more therapeutic agents simultaneously. Compared with single-agent treatments, these combination therapies often have enhanced efficacy, can delay the onset of resistance and may allow lower doses of individual drugs to be used, thus reducing toxicity. Multiple studies have investigated the potential of combining CDK7 inhibitors with other anticancer drugs [22, 23, 116, 124–127].

As ER is the key transcriptional driver of ER+ breast cancer and is activated by CDK7, CDK7 inhibitors have been assessed in combination with anti-oestrogens in this context (Table 3) [22, 125]. In the ER+ breast cancer cell line, MCF7, treatment with ICEC0942, plus either tamoxifen or fulvestrant, caused greater growth inhibition than either agent alone [22]. The combinatorial action of ICEC0942 with tamoxifen was also verified in mice bearing MCF7 xenografts [22]. In addition, ER-mutant breast cancer cells are especially sensitive to the combination treatment of THZ1 and fulvestrant [125]. ICEC0942 is now being assessed in combination with fulvestrant in clinical trials (Table 4).

It has been noted that some CDK7 inhibitors reduce expression of the anti-apoptotic BCL2 family member, MCL1 [23, 124, 127]; therefore, it is plausible that CDK7 inhibitors will work synergistically with apoptotic agents. CDK7 inhibitors have been investigated in combination with the apoptosis-inducing BH3-mimetics, venetoclax (ABT-199) [23, 124] and navitoclax (ABT-263) (Table 3) [127]. Venetoclax, which inhibits anti-apoptotic BCL2 to cause apoptosis, is approved for the treatment of CLL, small lymphocytic leukaemia (SLL) and AML [23]. The combination of SY-1365 and venetoclax is synergistic in AML cell lines and xenografts [23]. Although the current clinical trials of CDK7 inhibitors are focused on solid tumours, these results highlight the potential for CDK7 inhibitors to combat blood cancers, particularly if combined with BH3-mimetics. CDK7 inhibition also synergises with p53-activating agents, including the chemotherapeutic, 5-fluorouracil, to induce apoptosis in colorectal cancer cells [128].

BET inhibitors, compounds that target the bromodomain extra-terminal (BET) family of proteins, have garnered much recent interest as potential cancer therapeutics and are being assessed clinically across a range of cancers. The preclinical and clinical advancement of BET inhibitors in cancer therapy has recently been reviewed [141]. BET inhibitors are exemplified by the tool compound JQ1, which, like THZ1, preferentially represses SE-driven transcription and can be used to overcome oncogenic transcriptional addiction in cancer [19]. For this reason, THZ1 has been tested alongside JQ1 (Table 3), and this combination synergistically inhibits the growth of diffuse intrinsic pontine glioma (DIPG) [116], ovarian cancer [75] and neuroblastoma [126]. THZ1 has also been investigated alongside the histone deacetylase (HDAC) inhibitor, panobinostat (Table 3), for the treatment of the universally fatal paediatric cancer DIPG [116]. Like JQ1 and THZ1, panobinostat supresses transcription of SE-associated genes in DIPG and acts synergistically with CDK7 inhibition to suppress growth of DIPG-derived cell lines [116]. However, the development of CDK7 inhibitors with adequate brain penetrance would be required before this combination therapy could be achieved in the clinic.

Recent work has highlighted the potential of CDK7 inhibitors to be used in combination with immunotherapies. The CDK7 inhibitor, YKL-5-124, was shown to elicit immune response signalling in small cell lung cancer (SCLC), activating anti-tumourigenic T cells [131]. In immunocompetent mouse models of SCLC, the combination of YKL-5-124 and anti-PD-1 immune checkpoint inhibition increased overall survival in comparison with either treatment alone, and was further enhanced by the addition of chemotherapeutics (cisplatin and etoposide) [131].

Overall, there is much potential for CDK7 inhibitors to be used in combination with other drugs for cancer therapy. We anticipate that screening of CDK7 inhibitors in combination with other compounds, and large-scale genomic perturbation studies, may pave the way for the identification of additional co-targeting strategies.

### Resistance to CDK7 inhibitors

The emergence of resistance to cancer treatment, including targeted therapies, remains a major issue, and it is possible that even if CDK7 inhibitors prove a clinical success, resistance may develop in some patients. To gain understanding of potential resistance mechanisms, cell lines with acquired resistance to both ICEC0942 and THZ1 have been developed [142, 143].

ATP-binding cassette transporters (ABC-transporters) are a well-characterised mechanism of multidrug resistance in cancer and, when upregulated, can mediate the ATP-dependent efflux of drugs that are substrates. Upregulation of ABCB1, also known as p-glycoprotein, mediates resistance to THZ1 in neuroblastoma and lung cancer [142] and resistance to both THZ1 and ICEC0942 in breast cancer cell lines [143]. Increased expression of another ABC-transporter, ABCG2, results in resistance to THZ1 [142], but not to ICEC0942 [143]. With no clinical data available as yet, it remains to be seen whether ABC-transporter upregulation will arise in the context of CDK7 inhibitor resistance in patients. Despite numerous clinical trials, ABC-transport inhibitors have thus far proven unsuccessful in the clinic, mainly due to issues with potency and toxicity [144]. However, should future developments be made in this area, there may be scope to use CDK7 inhibitors in combination with ABC-transport inhibitors. Another way to overcome this mechanism of resistance is the development of CDK7 inhibitors that are not substrates for multidrug resistance transporters [145]. It is likely that other, as yet unidentified, mechanisms of resistance will also be important in a clinical setting.

As clinical trials progress, analyses of tumour features enriched in subsets of patients that are intrinsically resistant to CDK7 inhibitors, or those who acquire resistance after initially responding well, should help shed light on mechanisms of CDK7 inhibitor resistance. Alongside these, further preclinical models of CDK7 inhibitor resistance, including 3D cell culture and *in vivo* models, may prove informative. Ultimately, the identification of mechanisms of CDK7 inhibitor resistance should aim to aid the identification of patients who will derive most benefit from these drugs, helping to advance their clinical progress.

## Conclusions

CDK7 has a dual role in driving the cell cycle and transcription, is upregulated in a variety of cancers and has emerged as a promising cancer therapeutic target. At least ten selective inhibitors of CDK7, with activity against a wide range of cancer types, have been developed, their antitumour action likely mediated both through cell cycle arrest and inhibition of oncogenic transcriptional programs. In the preclinical setting, these inhibitors have demonstrated potential to overcome treatment-resistant cancer, both as monotherapies, and in combination with other cancer drugs. To date, four CDK7 inhibitors have progressed to Phase I/II clinical trial for the treatment of advanced solid malignancies. Whilst ABC-transporters can mediate resistance to some CDK7 inhibitors, additional factors that influence tumour response to CDK7 inhibition are yet to be identified. Further efforts to elucidate mechanisms of response, and to define patient selection strategies, will help to facilitate the clinical utility of CDK7 inhibitors.
